# Double whammy: the genetic variants in CECR2 and high Hcy on the development of neural tube defects

**DOI:** 10.3389/fgene.2023.1189847

**Published:** 2023-06-22

**Authors:** Baoling Bai, Qian Jiang, Lingyun Liu, Changyun Liu, Qin Zhang

**Affiliations:** ^1^Beijing Municipal Key Laboratory of Child Development and Nutriomics, Capital Institute of Pediatrics, Beijing, China; ^2^Department of Medical Genetics, Capital Institute of Pediatrics, Beijing, China; ^3^ Department of Pediatrics, Weifang Medical University, Weifang, Shandong, China

**Keywords:** mutation, homocysteine, neural tube defects, apoptosis, CECR2

## Abstract

**Introduction:** Neural tube defects (NTDs) are serious congenital malformations. The etiology of NTDs involves both genetic and environmental factors. Loss of CECR2 in mice has been shown to result in NTDs. Our previous study indicated that high homocysteine (HHcy) levels could further reduced the expression level of CECR2. This investigation aims to explore the genetic influence of the chromatin remodeling gene, *CECR2*, in humans and determine if HHcy can have a synergistic effect on protein expression.

**Methods:** We conducted Next-Generation Sequencing (NGS) of the *CECR2* gene in 373 NTD cases and 222 healthy controls, followed by functional assay application to select and evaluate *CECR2* missense variants and subsequent Western blotting to identify protein expression levels.

**Results:** From the analysis, we identified nine rare, NTD-specific mutations within the *CECR2* gene. Significantly, four missense variants (p.E327V, p.T521S, p.G701R, and p.G868R) were selected via functional screening. The E9.5 mouse ectodermal stem cell line NE-4C, transfected with plasmids expressing p.E327V, p.T521S, p.G868R variants or a recombinant harboring all four (named as 4Mut), exhibited notable reductions in CECR2 protein expression. Furthermore, exposure to homocysteine thiolactone (HTL), an extremely reactive homocysteine metabolite, amplified the reduction in CECR2 expression, accompanied by a significant increase in the apoptotic molecule Caspase3 activity, a potential NTD inducer. Importantly, folic acid (FA) supplementation effectively counteracted the CECR2 expression decline induced by CECR2 mutation and HTL treatment, leading to reduced apoptosis.

**Discussion:** Our observations underscore a synergistic relationship between HHcy and genetic variations in CECR2 concerning NTDs, thereby reinforcing the concept of gene-environment interaction phenomena in NTD etiology.

## Introduction

Neural tube defects (NTDs), which originate from the incomplete or failed closure of the neural tube during embryonic development, cause severe birth defects, including anencephaly, spina bifida and myelomeningocele (Greene and Copp, 2014). With a global prevalence ranging from 0.5 to >10 per 1,000 pregnancies (Avagliano et al., 2019; Peake et al., 2021), NTDs were the most predominant type of neonatal anomaly in low-income countries over the past decade (Anane-Fenin et al., 2023). To date, the strategy of intensifying preconception and antenatal supplementation of folic acid (FA), which has been implemented in a number of countries, has been pivotal in reducing NTDs (Blom et al., 2006). However, the existence of a subgroup of apparently folate-resistant NTDs, which account for an estimated one-third of NTDs, indicates that not all NTDs can be prevented with FA (Mosley et al., 2009; Blencowe et al., 2010). Therefore, we should clearly understand the causes of NTDs if we are to prevent them entirely.

Multiple complex factors are involved in the etiology of NTDs, and include genetic susceptibilities, environmental factors and unconscious genetic—environmental interactions (Copp and Greene, 2010; Greene and Copp, 2014). Elevation of maternal homocysteine (Hcy) levels (Yang et al., 2017), lower blood levels of the B-vitamin folate or inositol, maternal obesity and diabetes comprise the well-recognized environmental risk factors for NTDs. Notably, these environmental factors can also interact; for example, a deficiency of folate is considered to be the most common cause of high Hcy (HHcy) (Stanger et al., 2004; Copp et al., 2013). Additionally, with up to 70% of the variance in NTD prevalence being attributable to genetic factors (Leck, 1974), >200 candidate genes have been investigated and shown to induce NTDs (Harris and Juriloff, 2010). These include genetic polymorphisms such as C677T, and possibly A1298C, in the homocysteine remethylation gene that encodes methylenetetrahydrofolate reductase (MTHFR)*,* which may increase the risk of NTDs by approximately 1.8-fold (Boyles et al., 2005; Copp and Greene, 2010). Furthermore, genetic variants have the potential to interact with a predisposing environmental factor (e.g., maternal diabetes and folate status) to impact a NTD (Greene and Copp, 2014). For example, the folate one-carbon metabolism-related genes Mthfd1 and Folr1, and missense mutations of AMT or GLDC (Copp et al., 2013), have been associated with changes in maternal metabolism and secondary effects on the developing embryo. These observations prompted us to propose that there might be other genes with susceptibility to environmental factors associated with folate deficiency that also participate in the occurrence of NTDs.

CECR2 contains 19 exons and encodes a protein that contains both a DDT motif and a bromodomain, which is typical of proteins involved in chromatin remodeling (Banting et al., 2005). CECR2 is a critical member of the CECR2-containing remodeling factor (CERF) complex (Norton et al., 2022), an imitation switch (ISWI) chromatin-remodeling complex that is typically involved in DNA replication and repair, and transcriptional regulation. CECR2 has roles in neurulation, and the loss of CECR2 in mice can induce exencephaly (a perinatal-lethal cranial NTD), or lead to implantation failure or neonates with open eyelids (Banting et al., 2005; Thompson et al., 2012; Leduc et al., 2017). Additionally, 74% of BALB/c mice with a hypomorphic genetrap mutation (*Cecr2* ^*Gt45Bic*^) develop exencephaly, and *Cecr2* ^*tm1.1Hemc*^ mutants in both BALB/c and FVB/N strains show 96% penetrance for exencephaly (Fairbridge et al., 2010). Nevertheless, the specific role of CECR2 in neurulation is unknown.

We previously showed that maternal HHcy can result in decreased CECR2 transcription and the onset of NTDs (Zhang et al., 2018). Here, we have studied the effects of CECR2 variants found in human NTDs. Through screening of the exonic and highly conserved regions of CECR2 in 373 individuals with NTDs and 222 healthy controls, we identified nine NTD-specific rare variants in the NTD cohort. Four of these variants, all missense mutations, resulted in decreased expression of CECR2 and increased apoptosis in mouse cells. Furthermore, these effects were aggravated by the addition of HHcy treatment, but were attenuated by FA supplementation. Our study provides a mechanism for gene—environment interaction in NTDs.

## Materials and methods

### Human subjects

From 2005 to 2011, totally 373 Chinese Han individuals aged from gestational week (GW) 12 to 10-year-old with sporadic NTDs in this study were recruited and collected from multiple locations in the Northern area, including Shanxi, Liaoning, and Heilongjiang provinces (Li et al., 2020; Liu et al., 2018). Also Southern area of Tianjin and Jiangsu provinces were included. All patients with phenotypes of NTDs were diagnosed by clinical pathologists. In addition, a total of 222 ethnically and geographically matched subjects aged from GW14 to 18-year-old unrelated to NTD were recruited as controls. All subjects’ parents provided written informed consent as required by the Medical Ethics Committee of the Capital Institute of Pediatrics (Beijing, China) (Li et al., 2020). All studies were carried out in accordance with the principles of the Declaration of Helsinki.

### Genomic DNA sequencing

Genomic DNA was extracted from brain tissues of both individuals with NTDs and healthy controls using a Blood and Tissue DNA kit (Qiagen). The coding region and highly conserved region DNA of CECR2 gene were fragmented and enriched. The Truseq sample preparation kit (Illumina) was then used to prepare the sample according to the manufacturer’s standard protocol. The library is built from Agilent’s Custom sureelect Enrichment Toolkit (Agilent Technologies, Inc.) and Agilent’s Custom Enrichment Array. The hybridization reaction was performed on the AB 2720 Thermal cycle apparatus (Life Technologies). The reaction samples were incubated for 24 h in the hybrid mixture at 65°C and the lid was heated at 105°C. The SureDesign website uses Agilent technology to design custom captured oligonucleotides. The capture yield is enriched under the following cycle conditions:98°C 30 s; 10 cycles at 98°C for 10 s; 60°C 30 s; 72°C 30 s, 72°C 5 min. Base consolidation is then performed on a Genomic Analyzer II sequencer (Illumina). The sequencing results were consistent with UCSC GRCh37/hg19 with reference to the human genome was detected using Burrows-Wheeler Aligner v6.4. Polymerase chain reaction (PCR) repeats were sequenced and removed using Picard software. Single nucleotide variation (SNVS) was invoked using Genome Analysis Toolkit (GATK) and VarScan, and the SNVS were then labeled with ANNOVAR. Genotype calls were made using BayesAss 3.03 and identified variants were screened using dbSNP and Genome 1,000 browsers to identify shared variants in cases and controls. Sanger sequencing was performed to confirm missense mutations.

### Functional prediction of CECR2 variants

To predict the damage of 9 CECR2 variants to CECR2 protein function and structure, the Sorting Intolerant from Tolerant (SIFT) (http://sift-dna.org) (Sim et al., 2012) and PolyPhen-2 (http://genetics.bwh.harvard.edu/pph2/) algorithms were used for analysis the effect of coding variants on protein function. T-Coffee (https://www.ebi.ac.uk/Tools/msa/tcoffee/) was used for conservative analysis of amino acids.

### NE-4C cell culture, HTL and FA treatment

As described in our previous paper (Bai et al., 2018), NE-4C cells (ATCC number: SCRC-CRL-2925™) (Schlett and Madarasz, 1997) purchased from the Stem Cell Bank, Chinese Academy of Science. NE-4C were cultured on T25 cell culture dishes, which was pre-coated with 10 g/mL Poly-D-Lysine (Millipore) before 2 h passage. The complete medium composition is: 90% Eagle’s minimal essential medium (MEM) (ThermoFisher); 10% fetal bovine serum (FBS) (10099, Gibco); 1% GlutaMAX, and 1% non-essential amino acid.

To stimulate HHcy exposure, cells were pre-cultured in serum-free medium for 12 h, then 0.5 mM or 1 mM Homocysteine-thiolactone (HTL) (H6503, Sigma) was added to each group, 10% FBS was reintroduced, and cells were further cultured for 8 h for follow-up experiments. Cells without HTL treatment were used as a blank. In addition, FA (FA7876, sigma) is directly dissolved in the culture medium with or without HTL at 8 mg/L concentration.

### CECR2 plasmids and transfection

Wild-type (WT) human CECR2 and variant open-reading frames (ORFs), were synthesized by OriGene Technologies Co., Ltd. and were cloned into the expression vector pCMV6-AC. All WT and variant plasmids were validated by direct DNA sequencing.

A total of 10 µL of Lipofectamine^®^ 3,000 transfection reagent (L3000075, Thermo Fisher) diluted in 125 μL of Opti-MEM (51985-034, Thermo Fisher) was prepared. In addition, 5 μg of plasmid or 5 μg of empty vector were diluted with 125 μL of Opti-MEM and incubated for 15 min at room temperature. The 250 μL mixture of Lipo3000 and plasmids was added into one well. After incubated for 24–96 h, cells were harvested for further use in the following experiments. The grouping information is as follows: cells transfected empty vector as “NC” group; Cells transfected with the mutant are named according to the mutant type.

### Western blotting (WB)

Total cell lysate total proteins were separated by 4%-12% SDS-PAGE and subjected to WB assays with the primary antibody: anti-Cecr2 (PA5-82045, Thermofisher), anti-Cleaved Caspase-3 (Asp175) (#9661, CST). The data were standardized to the Gapdh (Anti-GAPDH, ab8245, abcam). Immunocomplexes were detected with a West Pico ECL kit (Thermo Scientific). The band intensities were determined using Image Lab software and expressed relative to Gapdh.

### Annexin-V and propidium iodide (PI) assays

To investigate the role of CECR2 mutants in apoptosis of NE-4C cells, we counted apoptotic cells under different culture conditions. After treatment, the cells were digested with EDTA-free trypsin, washed with cold PBS, and doubly stained with annexin-V and PI (KeyGen). Flow cytometric analysis was performed using the green fluorescent fluorescein isothiocyanate (FITC) channel tests annexin-V and red fluorescent for PI. The percentage of apoptotic cells in 10,000 cells was measured.

### Immunofluorescence (IF)

The cells were first washed twice with cold 1×PBS and treated as follows: fixed with 0.5% paraformaldehyde for 15 min, then permeated in 0.5%Triton X-100 at room temperature for 20 min, and blocked with 1 × PBS containing 10% normal goat serum and 0.3M glycine for 60 min. Cells were incubated with CECR2 antibody diluted in 5% normal goat serum overnight at 4°C. After washing, secondary antibodies with Alexa Fluor 488 (ab150077, Abcam) were incubated in the dark at room temperature for 1 h. The nuclei were restained with DAPI. Images were pictured on a Zeiss LSM710 confocal microscope.

### Statistical analysis

In the analysis of variation rate of human subjects, the difference between the case and control group was evaluated by Chi-square test or Fisher’s exact test. In the cell experiment, one-way analysis of variance (ANOVA) plus post-test was used to evaluate the statistical significance. All results are expressed as mean ± standard deviation (SD). All reported *p* values were 2 sides, *p* < 0.05 was considered statistically significant.

## Results

### Rare variants identified in CECR2

With the aim of identifying potential target genes for prevention of NTDs, we screened a total of 373 NTD cases and 222 healthy controls using next-generation capture target sequencing. This analysis identified nine nonsynonymous amino acid changes within the protein encoded by CECR2. All nine of the variants were found among 12 NTD patients and were absent in all 222 of the controls. The mutation rate of CECR2 was thus confirmed to be 3.22% (12/373) in the patients affected by NTDs. All variants were rare, with a frequency of <0.05% as reported by the Exome Aggregation Consortium (ExAC) database and the Genome Aggregation Database (gnomAD) (Table 1). All nucleotide changes were found in the heterozygous form.

**TABLE 1:** Variants identified in CECR2 in NTD patients through target gene NGS.

No.	Nucleotide change^a^	AA change^b^	Property change^c^	Conservation^d^	Domain^e^	Freq^f^	ExAC^g^	gnomAD^h^	SIFT^i^	PolyPhen-2b^j^
1	c.980A>T	p.Glu327Val	Acidic polar (negative) → nonpolar (neutral)	Yes	-	2/373	0.00004167	0.0000323	0.01	0.999
2	c.1126G>A	p.Val376Met	Nonpolar (neutral) → nonpolar (neutral)	No	**-**	1/373	0.0003	0.0002	0.05	0.990
3	c.1562C>G	p.Thr521Ser	Hydroxyl polar (neutral) → hydroxyl polar (neutral)	Yes	Bromo	1/373	-	-	0.01	0.740
4	c.1901A>T	p.His634Leu	Basic polar (positive) → nonpolar (neutral)	No	-	1/373	-	-	0.91	0.220
5	c.1940A>G	p.Gln647Arg	Amide polar (neutral) → basic polar (positive)	No	-	2/373	0.00008129	0.00009685	0.34	0.985
6	c.2101G>A	p.Gly701Arg	Nonpolar (neutral) → basic polar (positive)	Yes	-	1/373	-	-	0.10	1.000
7	c.2602G>A	p.Gly868Arg	Nonpolar (neutral) → basic polar (positive)	Yes	-	2/373	0.00004143	-	0.00	0.987
8	c.2822A>G	p.His941Arg	Basic polar (positive) → basic polar (positive)	No	-	1/373	0.000008287	-	0.88	0.201
9	c.3068C>T	p.Pro1023Leu	Nonpolar (neutral) → nonpolar (neutral)	No	-	1/373	0.0002	-	0.21	0.987

^a^ CECR2 GenBank RefSeq nos. NM_031413.3. Nucleotide numbering reflects cDNA, numbering with +1 corresponding to the A of the ATG, translation initiation codon 1 in the reference sequence.

^b^ The position of the mutations is given with reference to sequence accession NP_113601.2 for the protein.

^c^ Amino acid residue property change.

^d^ Amino acid residue evolutionary conservation.

^e^ Location in protein secondary structure.

^f^ Number of mutation carriers in NTD, cases.

^g^ The Exome Aggregation Consortium: http://exac.broadinstitute.org.

^h^ The Genome Aggregation Database: http://gnomad-sg.org.

^i^ Score ranges from 0-1, if the score is ≤ 0.05, the amino acid substitution was predicted to be damaging.

^j^ Score ranges from 0-1, the closer the score is to 1, the greater the probability that amino acid replacement will damage the structure and function of protein.

**FIGURE 1:**
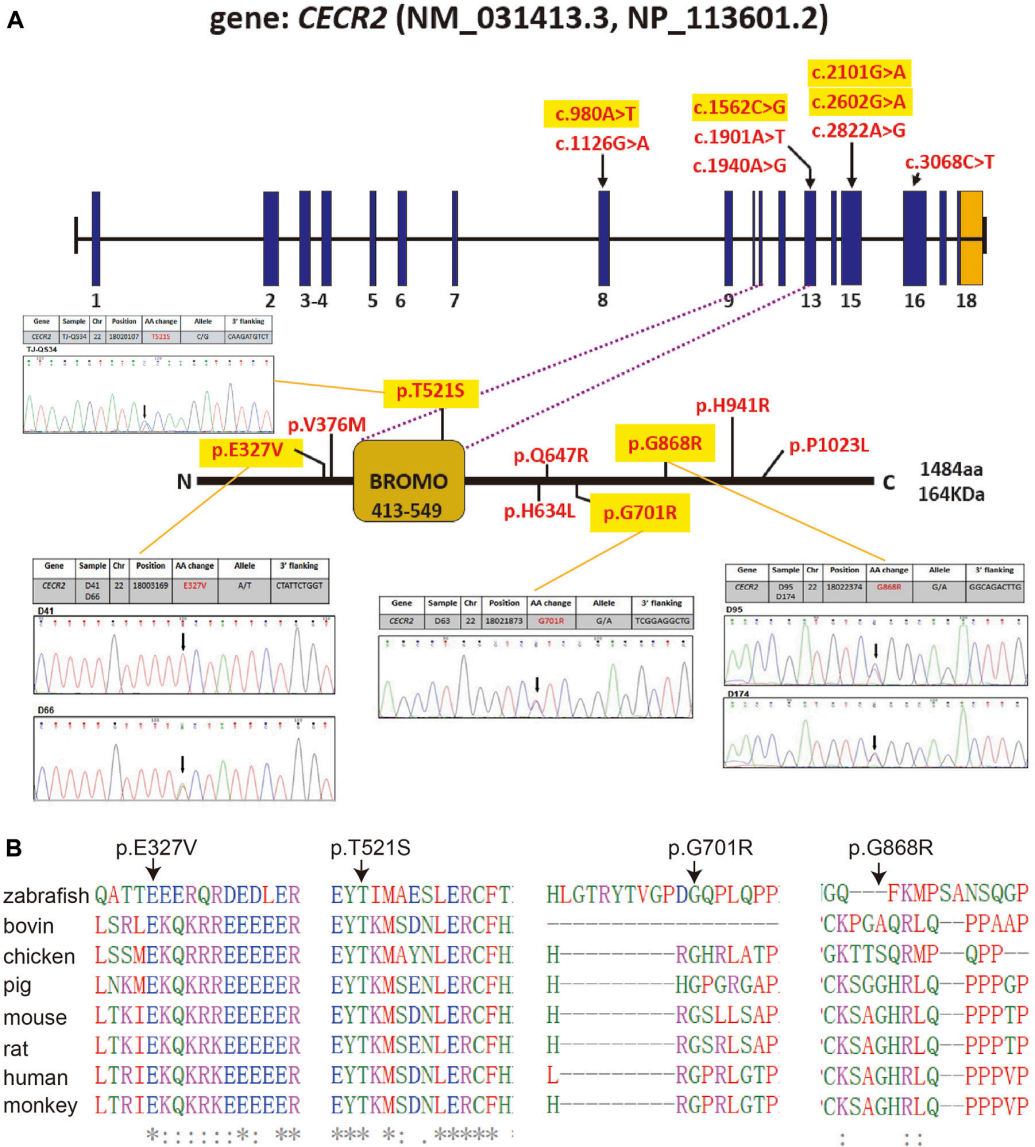
Rare missense variants identified in CECR2 gene. **(A)**Structure of the CECR2 transcript (NM_031413.3) and proteins (NP_113601.2), positions of 9 identified missense variants were marked in the gene sequence. Heterozygous variants of p.Glu327Val (c.980A>T), p.Thr521Ser (c.1562C>G), p.Gly701Arg (c.2101G>A) and p.Gly868Arg (c.2602G>A) were further confirmed via sequencing. **(B)** Alignment of human CECR2 protein sequence with other orthologues sequences, including zebrafish: A0A8M1RL92 (Uniprot); bovin: F1MRJ9; chicken: A0A8V0YBW6; pig: A0A287AW08; mouse: E9Q2Z1; rat: F1LYI0; and monkey: A0A2K5EPY0 by T-Coffee. Solid black arrow indicate a variant.

The nine nonsynonymous variants detected in NTD cases were as follows: p.Glu327Val (c.980A>T), p.Val376Met (c.1126G>A), p.Thr521Ser (c.1562C>G), p.His634Leu (c.1901A>T), p.Gln647Arg (c.1940A>G), p.Gly701Arg (c.2101G>A), p.Gly868Arg (c.2602G>A), p.His941Arg (c.2822A>G) and p.Pro1023Leu (c.3068C>T) (Figure 1). Among these missense changes, p.Thr521Ser was located in the bromodomain. Amino acid conservation analysis showed that four of these mutations (p.Glu327Val, p.Thr521Ser, p.Gly701Arg and p.Gly868Arg) affected highly conserved amino acid residues, while the other five involved less conserved amino acid residues (Table 1). On the basis of bioinformatics, including analysis of amino acids property changes, conservation, localization in vital domains, and damage to protein structure or function using the SIFT/PolyPhen-2 algorithm, we selected the following four mutations for functional studies: p.Glu327Val (c.980A>T), p.Thr521Ser (c.1562C>G), p.Gly701Arg (c.2101G>A) and p.Gly868Arg (c.2602G>A).

### Clinical features of NTD patients carrying CECR2 mutations

Six patients carrying a mutation at one of the four selected sites presented varying clinical features of NTDs. The p.Gly868Arg variant was detected in two patients, p.Glu327Val was found in two patients, and both of the remaining two mutations had only one carrier each. The only CECR2 mutation that was located in the bromodomain of the protein, p.Thr521Ser, was identified in a 4-year-old male patient (TJ-QS34) who was affected by a C2-3 vertebral fusion, an untypical NTD phenotype. The CECR2 p.Glu327Val (c.980A>T) substitution was found in two female fetuses (D41 and D66). D41 presented with congenital hydrocephalus and open lumbosacral spina bifida at 20 weeks of gestation. D66 presented with anencephaly and open spina bifida at 17 weeks of gestation, and was also affected by atelectasis, visceral congestion and single umbilical artery. A male patient (D63) carrying the CECR2 p.Gly701Arg (c.2101G>A) variant also presented with anencephaly at 25 weeks of gestation, and was affected by multiple abnormalities, including meningoencephalocele, atelectasis, visceral congestion, cheilopalatognathus, symphysodactylia, equinovarus and absence of the eyes and nose. Two female fetuses (D95 and D174) had the same mutation, CECR2 p.Gly868Arg (c.2602G>A). Although both patients presented with spina bifida, there were phenotypic variations: D95 had open occipitocervical thoracolumbar spina bifida, while D174 had non-open thoracolumbar spina bifida. In addition to spina bifida and other systemic defects, D95 was also affected by anencephaly, the most severe type of NTD, while D174 was affected by congenital hydrocephalus, which is commonly associated with spina bifida (Table 2).

**TABLE 2:** Clinical characteristics and molecular findings in patients with neural tube defects^a^.

**Patient**	D41	D66	TJ-QS34	D63	D95	D174
Validated mutations^b^ (protein alteration (cDNA))	p.Glu327Val (c.980A>T)		p.Thr521Ser (c.1562C>G)	p.Gly701Arg (c.2101G>A)	p.Gly868Arg (c.2602G>A)	
Gender	F	F	M	M	F	F
Ethnicity	Northern Chinese	Northern Chinese	Southern Chinese	Northern Chinese	Northern Chinese	Northern Chinese
Gestational age (weeks)	20	17	4Y^c^	25	21	17
Clinical features						
Anencephaly	−	+	−	+	+	−
Congenital hydrocephalus	+	−	−	−	−	+
Spina bifida	+^d^	+^e^	−	−	+^f^	+^g^
Meningoencephalocele	−	−	−	+	−	−
Atelectasis	−	+	−	+	−	+
Visceral congestion	+	+	−	+	−	+
Mild placental hemorrhage	−	−	−	+	−	+
C2-3 Vertebral fusion	−	−	+	−	−	−
Absence of eye and nose	−	−	−	+	−	−
Cheilopalatognathus	−	−	−	+	−	−
Symphysodactylia	−	−	−	+	−	−
Single umbilical artery	−	+	−	−	−	−
Equinovarus	−	−	−	+	−	−

^a^ This table summarizes the clinical findings in the study participants.

^b^ The numbering of the mutations and alterations is relative to NM_031413.3 (gene) and NP_113601.2 (protein), respectively.

^c^ The patient is 4 years old.

^d^ Open lumbarsacral spina bifida.

^e^ Open spina bifida.

^f^ Open occipitocervical thoracolumbar spina bifida.

^g^Non-open thoracolumbar spina bifida.

### Mutations of CECR2 affected protein expression of CECR2

**FIGURE 2:**
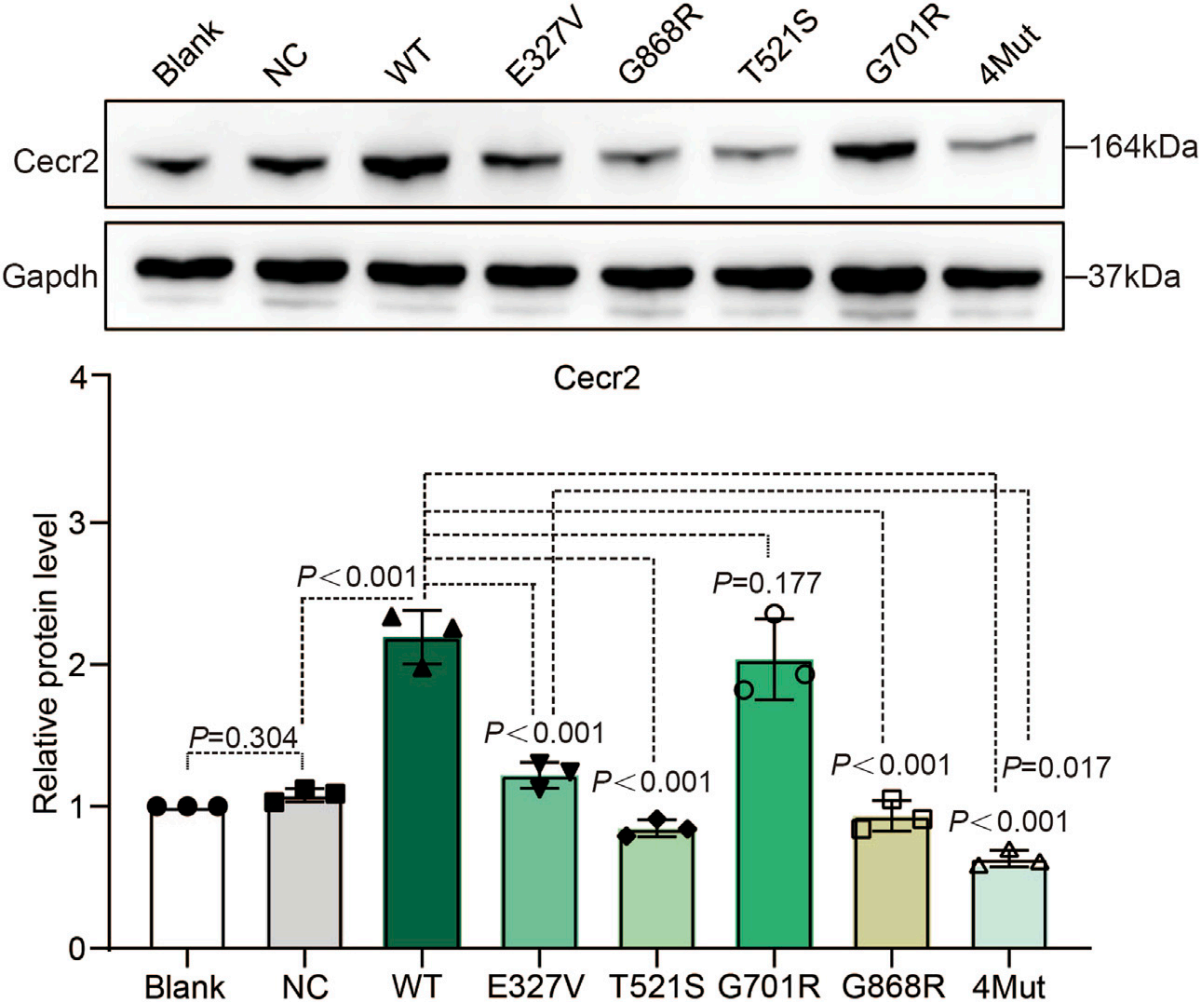
Effects of CECR2 missense variants on CECR2 protein expression. NE-4C cells were transfected with wild-type (WT), empty vector plasmid, or the 4 selected CECR2 variants. WB was performed with whole-cell proteins lysate and analyzed proteins were immunoblotted with anti-Cecr2 antibody (Upper), and anti-Gapdh antibody (lower), which was served as loading control.

Given that reduction of Cecr2 in mice resulted in lethal exencephaly (Leduc et al., 2017; Niri et al., 2021), we addressed the question of whether the four CECR2 rare missense variants found in human patients would affect CECR2 protein expression *in vitro*. A wild-type (WT) CECR2 expression plasmid (WT group) and an empty control plasmid (NC group) were constructed and transiently transfected into NE-4C, a mouse E9 neural ectoderm cell line (Demeter et al., 2004). Western blotting assays revealed that protein levels of CECR2 did not differ between the NC group and the Blank group (normally cultured cells), but were remarkably higher in the WT group. We also constructed expression plasmids for each of the variants, and presumed that the quality of transfection of each expression plasmid would be equivalent. Expression levels of the p.E327V, p.T521S and p.G868R variants of CECR2 were significantly lower than that of WT group; notably, p.T521S led to a particularly significant decrease in total CECR2 expression (Figure 2). By contrast, expression of the p.G701R variant was comparable to that of WT, indicating that this mutation did not affect protein expression of CECR2. Furthermore, cells transfected with a recombinant variant containing all four mutations (4Mut) of CECR2 showed significantly decreased expression compared with WT CECR2 or the p.E327V single mutant, suggesting that multiple mutations could worsen stability of the protein.

### The p.T521S variant and HHcy synergistically decreased CECR2 expression

**FIGURE 3:**
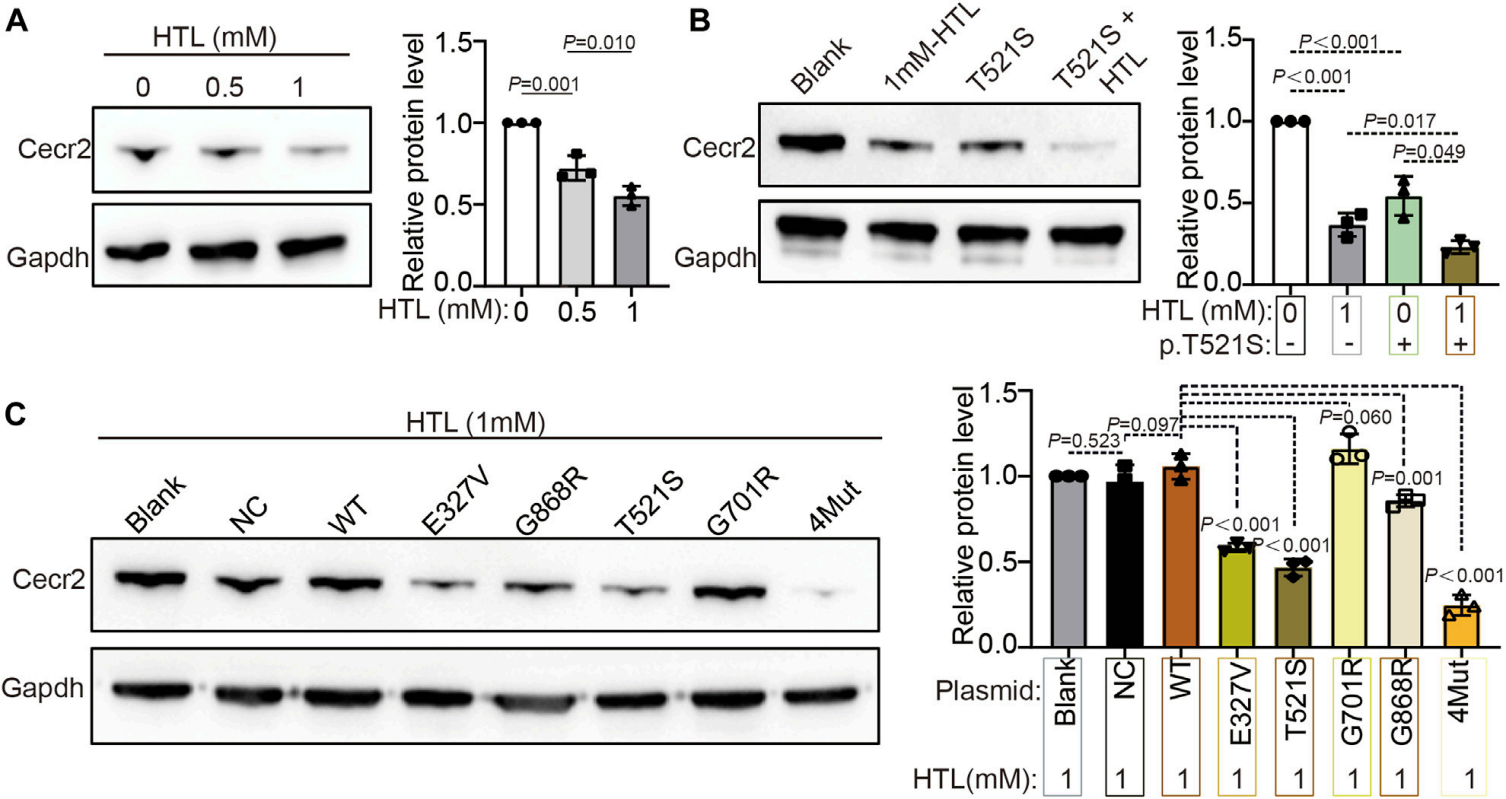
The HHcy and CECR2 missense variants synergistically reduced the expression of CECR2 protein expression. **(A)** NE-4C were treated with different concentrations of HTL, and the expression level of CECR2 was detected by WB. **(B)** Transfected the mutant p.T521S into NE-4C cells with or without 1 mM HTL treatment, and detect the effect on the expression of Cecr2 protein by WB. **(C)** NE-4C cells were treated with 1 mM HTL and transfected with 4 CECR2 variants, respectively. WB was further employed to detect CECR2 protein expression.

NTDs are the result of interactions between genes and environment. On the basis of our previous research on the reduction in CECR2 gene expression induced by HHcy (Zhang et al., 2018), we wondered whether the genetic variation in Cecr2 and HHcy might have a synergistic effect on the expression of CECR2. First, by employing Western blotting analysis, we confirmed that CECR2 protein expression was indeed downregulated in NE-4C cells treated with HTL, a highly reactive Hcy metabolite (Mei et al., 2020). Furthermore, the reduction in CECR2 expression was stronger in cells treated with 1 mM HTL than in those treated with 0.5 mM HTL, demonstrating a dose-dependent effect of HTL (Figure 3A). Next, we examined the effect of HTL treatment on CECR2 expression in cells transfected with the p.T521S plasmid. The expression of CECR2 in the p.T521S + 1 mM HTL was significantly lower than that in the HTL-treated group or the untreated p.T521S group, suggesting that the p.T521S mutant and HHcy could work synergistically to reduce CECR2 protein expression (Figure 3B). Additionally, to further demonstrate that HTL can aggravate the disruption of CECR2 protein expression, we transfected in 1 mM HTL-treated cells with WT, p.E327V, p.G701R or p.G868R, or 4Mut plasmid. As shown in Figure 3C, compared with the Blank + HTL and NC + HTL groups, CECR2 expression did not change significantly in the WT + HTL group, suggesting that, in cells carrying a CECR2 overexpression plasmid, simultaneous HTL treatment can restore the normal expression of CECR2. However, in cells treated with p.E327V + HTL, p.G868R + HTL or 4Mut + HTL, the expression levels of CECR2 were still significantly lower than that in the WT + HTL group, which was consistent with the results in Figure 2. Collectively, these cell-based results demonstrated that HTL can co-regulate CECR2 expression with the p.T521S, p.E327V or p.G868R mutants of CECR2.

### The p.T521S variant cooperated with HHcy to induce increased apoptosis of NE-4C cells

**FIGURE 4:**
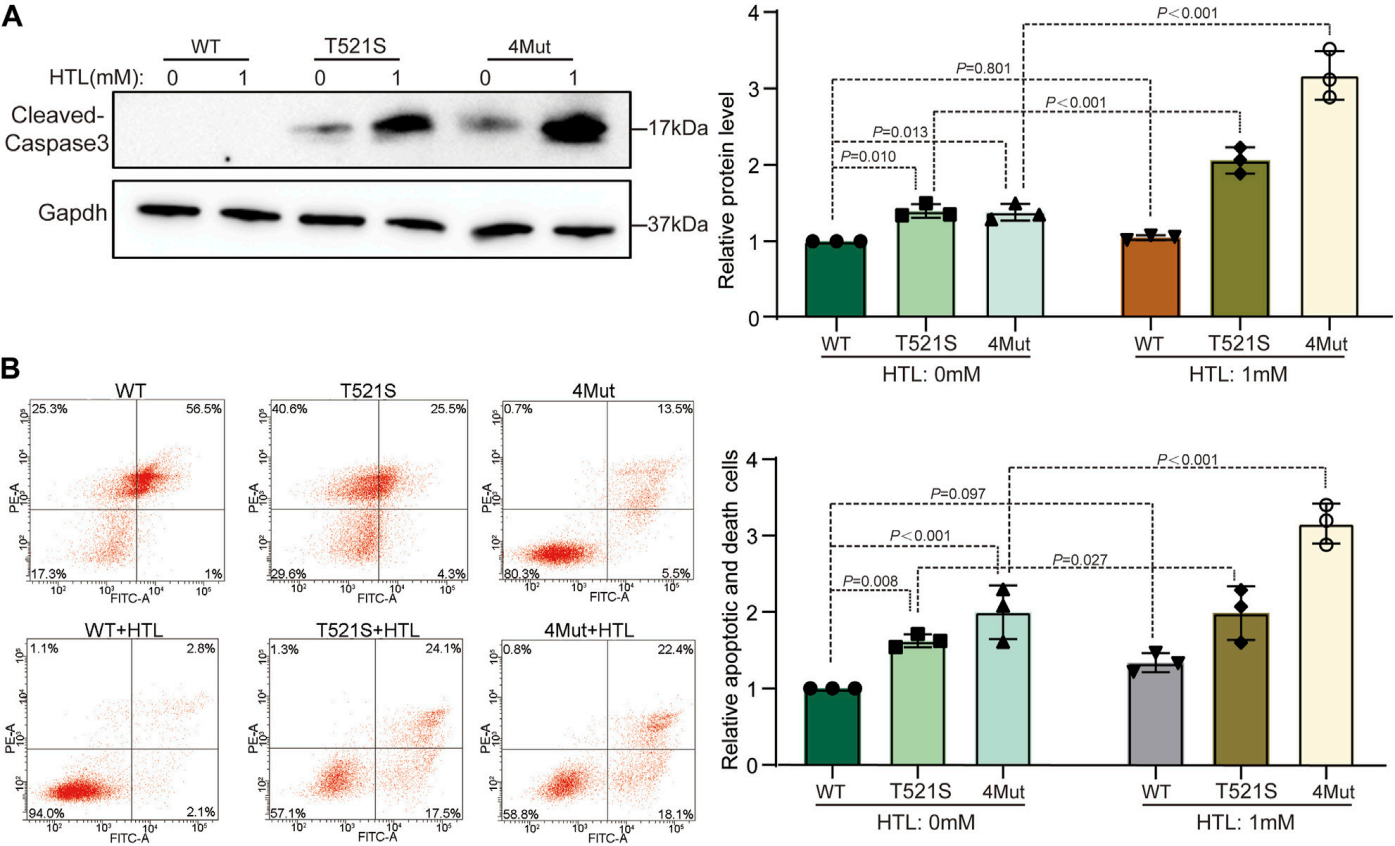
The p.T521S variant induce cells apoptosis. **(A)** After transfection with p.T521S mutant or 4Mut plasmids, the expression level of apoptotic molecules: cleaved-caspase3 was detected by WB. **(B)** Cell apoptosis was analyzed by flow cytometry after cells double staining with FITC and PI.

Given that an increase in apoptosis is one of the most common cellular changes observed in studies of gene-targeted embryos that produce NTDs (Copp and Greene, 2010), we aimed to examine expression of cleaved caspase-3, a molecule related to both apoptosis and NTDs (Houde et al., 2004; Zhou et al., 2018), in cells transfected with the Cecr2 mutants. As shown in Figure 4A, the p.T521S-transfected cells exhibited enhanced apoptosis compared to that in WT-transfected cells. Additionally, 1 mM HTL treatment combined with p.T521S transfection led to stronger enhancement of cleaved caspase-3 expression than p.T521S transfection alone. This trend of increased cleaved caspase-3 expression was also found in cells transfected with 4Mut. To test for the possibility of impairment of apoptosis at the cellular level, we performed annexin V and propidium iodide (PI) assays. Consistent with the increased cleaved caspase-3 activity, the percentage of apoptotic cells was decreased in both the p.T521S and 4Mut transfectants compared with that in the WT transfectants. By contrast, when the cultures of p.T521S or 4Mut transfectants were superimposed with HTL treatment, the percentages of apoptotic cells increased significantly compared with untreated cultures (*p* < 0.05; Figure 4B). Taken together, these data suggested that the decrease in CECR2 protein resulting from CECR2 gene mutation or co-treatment with HTL led to cell apoptosis.

### FA supplementation rescued the reduction in CECR2 caused by CECR2 variants, with or without HHcy

**FIGURE 5:**
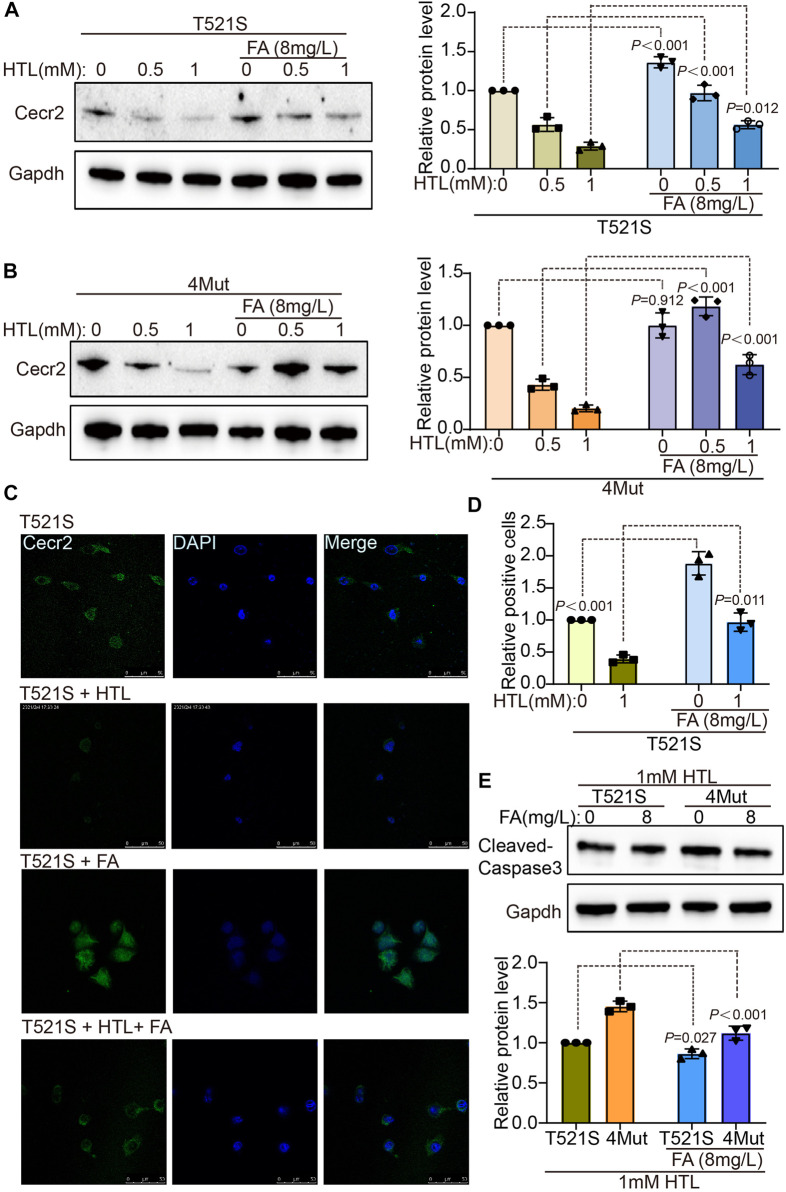
Folic acid can rescue decreased of CECR2 protein expression caused by CECR2 missense variants or high hcy. The cells treated with HTL were transfected with p.T521S variant **(A)** or 4-mixed variant **(B)**, respectively, and supplemented with or without FA, the expression of Cecr2 protein under different treatment conditions was detected by WB method. **(C,D)** IF assays were performed to detect the effect of FA on the expression and distribution of Cecr2 protein in p.T521S variant transfected cells. **(E)** The rescue effect of FA on apoptosis was detected by WB in p.T521S or 4Mut transfected cells.

FA supplementation is effective in preventing the occurrence of NTDs (Crider et al., 2022), leading us to wonder whether FA could compensate for the abnormal CECR2 expression caused by the genetic variation in CECR2 in patients with NTDs. First, we examined the effect of 8 mg/L FA supplementation on cells transfected with the p.T521S variant, with or without co-treatment with 0.5 mM HTL or 1 mM HTL. As shown in Figures 5A, B, FA supplementation significantly increased the expression level of CECR2 in all three treatment groups. Additionally, FA supplementation had the almost same effect on cells transfected with 4Mut plasmid. To further elucidate the distribution and expression of CECR2 at the cellular level, we used the IF method to detect cells transfected with p.T521S and superimposed with different treatments. As shown in Figures 5C, D, CECR2 was mainly expressed in the cell nucleus. Compared with the p.T521S-transfected group, CECR2 expression was significantly downregulated in the p.T521S + HTL group. Supplementation of FA in both of these groups effectively increased the expression of CECR2 in the nucleus. FA supplementation also significantly downregulated cleaved caspase-3 expression in p.T521S- or 4Mut-transfected cells superimposed with HTL treatment (Figure 5E), suggesting that FA effectively reversed the damage caused by CECR2 variants and HTL treatment.

## Discussion

In this study, we correlated CECR2 gene mutations and HHcy with CECR2 protein expression in NE-4C cells using integrative analysis of Western blotting and PI assay data. Our results indicated that three specific mutations identified in human NTDs (p.E327V, p.T521S, and p.G868R) led to reduced CECR2 protein expression, which further induced cell apoptosis. We propose that the increased cell apoptosis might have been responsible for the failure of neural tube closure in these patients. These effects were more severe when combined with HHcy treatment, but were effectively rescued by FA supplementation. Our results provide new evidence for the interaction between gene variants and environmental factors involved in the NTDs.

In our prior study, we identified four rare mutations in the CASPASE9 gene across 7 NTD samples and eight rare mutations in the DNAAF1 gene from 9 NTD patients (Miao et al., 2016; Liu et al., 2018). In this current research, we detected 9 point mutations in the CECR2 gene within 12 samples among 373 NTD cases. These low-frequency genetic variants alone may not directly cause NTDs; however, the simultaneous presence of multiple gene variants could have a synergistic effect on the occurrence of NTDs. For instance, variants in the CYP26B1 gene, occurring concurrently with other variants in neural tube-related genes such as CELSR3 and REST, were hypothesized to be associated with the craniorachischisis phenotype (Zou et al., 2020). The risk of NTDs was three times greater in subjects carrying both heterozygous MTHFR C677T and MTR A2756G genotypes compared to those with wild-type homozygous AA and CC genotypes (Kumari et al., 2022). Moreover, an accumulation of approximately nine SloFVs (singleton loss-of-function variants) represents a genomic threshold for NTD risk, regardless of genetic background or ethnicity (Chen et al., 2018). These findings emphasize the importance of investigating low-frequency gene point mutations for identifying high-risk NTD groups, which may offer targeted sites for clinical personalized therapy.

Reduction or loss of CECR2 in mice led to lethal exencephaly (Leduc et al., 2017). Several point mutations in CECR2 that also resulted in exencephaly include mice with *Cecr2* ^*tm1.1Hemc*^ and the hypomorphic genetrap mutation *Cecr2* ^*Gt45Bic*^ (Fairbridge et al., 2010). Among the six clinical NTD cases with CECR2 variants in our cohort (Table 2), the most common NTD phenotypes were spina bifida and anencephaly (exencephaly), and these patients also exhibited meningoencephalocele, hydrocephalus and other deformities, suggesting that the NTD phenotype associated with CECR2 mutation may be diverse.

Verification of the protein expression of the CECR2 mutants in the NE-4C cell model (Figure 2) showed that three of the variants (p.E327V, p.T521S, and p.G868R) exhibited significantly lower expression compared with WT, and one did not (p.G701R). Notably, the 4Mut variant showed a greater reduction in CECR2 protein than the single-point mutant p.E327V. Previous reports showed that *Cecr2* ^*Gt45Bic*^ homozygous mutants had a wider distance between the cranial neural folds, while *Cecr2* ^*Gt45Bic*^ heterozygous embryos had a slight delay in neural tube closure (Dawe et al., 2011). Together, these data suggested that the CECR2 variants may have a degree of dosage sensitivity.

At present, the molecular mechanism of NTDs induced by reduction of CECR2 protein is unclear. Despite its DNA repair function, CECR2 was not required for double-strand break repair in primary neurospheres (Elliott et al., 2020). In a study using embryonic stem cells, CCAR2, LUZP1, and the ISWI proteins SMARCA5 and SMARCA1 were found to combine with CECR2 to structure and stabilize CERF components (Niri et al., 2021). Although no studies have reported on the involvement of these ISWI proteins in CECR2 variant-related NTDs, in the 11–14 somite stage, *Cecr2* ^*Gt45Bic*^ mutant embryos exhibited an approximately 7- to 14-fold reduction in CECR2 transcription from normal levels. This reduction resulted in downregulation of Alx1/Cart1 and Dlx5, which when mutated can result in exencephaly in mice (Fairbridge et al., 2010), indicating that CECR2 mutants may be involved in NTDs by inducing downstream changes in gene expression. This phenomenon was also observed in our study: the NTD-related gene encoding caspase-3 (Urase et al., 2003; Houde et al., 2004) was abnormally activated in the p.T521S and 4Mut CECR2 mutants (Figure 4).

Our results also highlighted the interaction between Hhcy, an environmental factor, and CECR2 mutations in the inhibition of CECR2 and activation of apoptosis. Their destructive effects were more serious than those caused by the single CECR2 mutants or HHcy treatment alone, indicating a superimposition effect of HHcy and CECR2 variation, which was effectively attenuated by FA supplementation (Figures 3–5). Both folate deficiency and HHcy are environmental factors for increased risk of NTDs (Zhang et al., 2018; Wang et al., 2021; Wang et al., 2023). Furthermore, folate deficiency can lead to HHcy through one-carbon metabolism (Bhatia and Singh, 2015; Kaye et al., 2020), implying a crosslink between these two factors. Our results also suggested that the two had a synergistic regulatory effect on CECR2, in which the CECR2 mutants participated. Additionally, the interaction between HHcy and variation of the MTHFR gene, including the C677T and A1298C mutations, was shown to increase susceptibility to vascular diseases and neural tube defects in the progeny (Pawlak and Strauss, 2001). Taken together, our data expanded the previous results, emphasizing the increased susceptibility to NTDs by the synergistic regulatory effect of HHcy and CECR2 mutation.

In this study, we identified and described nine rare mutations of the CECR2 gene in NTD cases and demonstrated that three of the variants (p.T521S, p.E327V and p.G868R) impaired protein expression, suggesting loss-of-function variation. We also showed that the p.T521S variant activated apoptosis, which became more serious under HHcy conditions. Our findings confirm the association of apoptosis with human NTDs and highlight the effect of gene—environment interactions in this complex disease.

## Data Availability

The original contributions presented in the study are included in the article/supplementary material, further inquiries can be directed to the corresponding authors.
